# 5,5-Bis(hydroxy­meth­yl)-2-phenyl-1,3-dioxane

**DOI:** 10.1107/S1600536808022046

**Published:** 2008-07-19

**Authors:** Yi-Ming Luo, Xing-Ming Liu, Xian-You Yuan, Min Zhang, Seik Weng Ng

**Affiliations:** a School of Chemistry and Chemical Engineering, Central South University, Changsha 410083, People’s Republic of China; b Department of Biology and Chemistry, Hunan University of Science and Engineering, Yongzhou 425100, People’s Republic of China; c Department of Chemistry, University of Malaya, 50603 Kuala Lumpur, Malaysia

## Abstract

In the title compound, C_12_H_16_O_4_, the 1,3-dioxane ring adopts a chair conformation; the 2-phenyl substitutent occupies an equatorial position. Adjacent mol­ecules are linked by O—H⋯O hydrogen bonds into a chain.

## Related literature

For the crystal structures of similar 5-aryl-1,3-dioxanes, see: Al-Mughaid *et al.* (2003 ▶); Grosu *et al.* (1997 ▶, 1998 ▶). For applications of this class of compounds, see: Wang *et al.* (1994 ▶); Yuan *et al.* (2005 ▶).
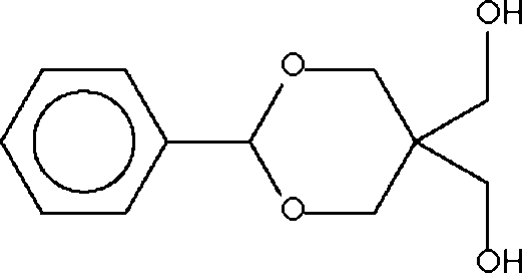


## Experimental

### 

#### Crystal data

C_12_H_16_O_4_
*M* *_r_* = 224.25
Orthorhombic, 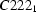

*a* = 6.2654 (4) Å
*b* = 10.4593 (6) Å
*c* = 34.5285 (19) Å
*V* = 2262.7 (2) Å^3^
*Z* = 8
Mo *K*α radiation
μ = 0.10 mm^−1^
*T* = 173 (2) K
0.46 × 0.42 × 0.21 mm

#### Data collection

Bruker SMART 1000 diffractometer
Absorption correction: none
5873 measured reflections
1403 independent reflections
1340 reflections with *I* > 2σ(*I*)
*R*_int_ = 0.031

#### Refinement

*R*[*F* ^2^ > 2σ(*F* ^2^)] = 0.066
*wR*(*F* ^2^) = 0.184
*S* = 1.14
1403 reflections
145 parameters
H-atom parameters constrained
Δρ_max_ = 0.25 e Å^−3^
Δρ_min_ = −0.41 e Å^−3^

### 

Data collection: *SMART* (Bruker, 1997 ▶); cell refinement: *SAINT* (Bruker, 2003 ▶); data reduction: *SAINT*; program(s) used to solve structure: *SHELXS97* (Sheldrick, 2008 ▶); program(s) used to refine structure: *SHELXL97* (Sheldrick, 2008 ▶); molecular graphics: *X-SEED* (Barbour, 2001 ▶); software used to prepare material for publication: *publCIF* (Westrip, 2008 ▶).

## Supplementary Material

Crystal structure: contains datablocks global, I. DOI: 10.1107/S1600536808022046/wn2272sup1.cif

Structure factors: contains datablocks I. DOI: 10.1107/S1600536808022046/wn2272Isup2.hkl

Additional supplementary materials:  crystallographic information; 3D view; checkCIF report

## Figures and Tables

**Table 1:** Hydrogen-bond geometry (Å, °)

*D*—H⋯*A*	*D*—H	H⋯*A*	*D*⋯*A*	*D*—H⋯*A*
O4—H4O⋯O3^i^	0.84	2.19	2.644 (6)	114

Symmetry code: (i) 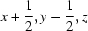.

**Fig. 1.**
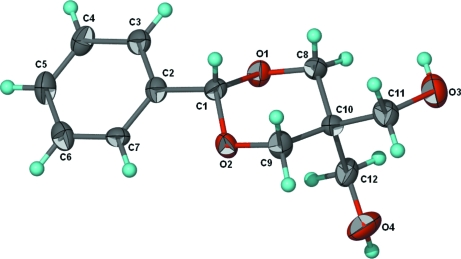
The molecular structure of the title compound, with displacement ellipsoids drawn at the 70% level. Hydrogen atoms are drawn as spheres of arbitrary radius.

## supplementary crystallographic information

### Comment

The title compound was synthesized to be used as a precursor in organic syntheses. This class of compounds has useful insecticidal as well as anti-foaming properties (Yuan *et al.*, 2005; Wang *et al.*, 1994). The 1,3-dioxane ring adopts a chair conformation; the phenyl substitutent in the 2-position occupies an equatorial position (Fig. 1). Adjacent molecules are linked by O—H···O hydrogen bonds into a chain. The crystal structures of some similar 5-aryl-1,3-dioxanes have been reported (Al-Mughaid *et al.*, 2003; Grosu *et al.* 1997; 1998).

### Experimental

2,2-Bis(hydroxymethyl)-1,3-propanediol (4.0 g, 30 mmol) and DMF (20 ml) were heated to 353 K. Iodine (0.5 g in an active carbon load of 23.6% by mass) and benzaldehyde (30 ml) were added. The clear solution was heated at 353–363 K for 5 h. The solution was filtered hot and the solvent removed by evaporation. The residue was dissolved in diethyl ether (50 ml) and the solution washed with 5% aqueous sodium bicarbonate. The diethyl ether solution was dried over sodium sulfate. Removal of the solvent gave a solid that was recrystallized from ethyl acetate (yield 5.5 g, 80%); m.p. 408 K.

### Refinement

In the absence of significant anomalous scattering effects, Friedel pairs were merged. Carbon-bound H-atoms were placed in calculated positions (C—H 0.95 to 0.99 Å) and were included in the refinement in the riding model approximation, *U*_iso_(H) = 1.2*U*_eq_(C). The H-atoms of the hydroxyl groups were placed at calculated positions and then refined as riding; O—H = 0.84 Å and *U*_iso_(H) = 1.5*U*_eq_(O). For one of the two hydroxyl groups (O3), its hydrogen atom does not form a hydrogen bond to an adjacent acceptor atom. Other possibilities for placing hydrogen atoms on the two groups led to unacceptably short H···H interactions of less than 2 Å.

### Crystal data

**Table:**

C_12_H_16_O_4_	*F*_000_ = 960
*M**_r_* = 224.25	*D*_x_ = 1.317 Mg m^−^^3^
Orthorhombic, *C*222_1_	Mo *K*α radiation λ = 0.71073 Å
Hall symbol: C 2c 2	Cell parameters from 4610 reflections
*a* = 6.2654 (4) Å	θ = 2.4–27.0º
*b* = 10.4593 (6) Å	µ = 0.10 mm^−^^1^
*c* = 34.5285 (19) Å	*T* = 173 (2) K
*V* = 2262.7 (2) Å^3^	Block, colorless
*Z* = 8	0.46 × 0.42 × 0.21 mm

### Data collection

**Table:**

Bruker SMART 1000 diffractometer	1340 reflections with *I* > 2σ(*I*)
Radiation source: fine-focus sealed tube	*R*_int_ = 0.031
Monochromator: graphite	θ_max_ = 27.0º
*T* = 173(2) K	θ_min_ = 2.4º
φ and ω scans	*h* = −7→7
Absorption correction: None	*k* = −13→11
5873 measured reflections	*l* = −24→44
1403 independent reflections	

### Refinement

**Table:**

Refinement on *F*^2^	Secondary atom site location: difference Fourier map
Least-squares matrix: full	Hydrogen site location: inferred from neighbouring sites
*R*[*F*^2^ > 2σ(*F*^2^)] = 0.066	H-atom parameters constrained
*wR*(*F*^2^) = 0.184	*w* = 1/[σ^2^(*F*_o_^2^) + (0.059*P*)^2^ + 10.1519*P*] where *P* = (*F*_o_^2^ + 2*F*_c_^2^)/3
*S* = 1.14	(Δ/σ)_max_ = 0.001
1403 reflections	Δρ_max_ = 0.25 e Å^−^^3^
145 parameters	Δρ_min_ = −0.41 e Å^−^^3^
Primary atom site location: structure-invariant direct methods	Extinction correction: none

### Fractional atomic coordinates and isotropic or equivalent isotropic displacement parameters (Å^2^)

**Table:**

	*x*	*y*	*z*	*U*_iso_*/*U*_eq_	
O1	0.8999 (6)	0.8806 (3)	0.63367 (9)	0.0263 (8)	
O2	0.7606 (6)	0.6825 (3)	0.61705 (10)	0.0271 (8)	
O3	0.3975 (7)	0.9308 (4)	0.71647 (11)	0.0410 (10)	
H3O	0.4584	0.9956	0.7073	0.061*	
O4	0.6297 (7)	0.6260 (4)	0.71881 (12)	0.0509 (12)	
H4O	0.7073	0.5941	0.7361	0.076*	
C1	0.8416 (8)	0.7994 (4)	0.60313 (13)	0.0223 (10)	
H1	0.7304	0.8424	0.5869	0.027*	
C2	1.0363 (7)	0.7728 (4)	0.57852 (12)	0.0216 (9)	
C3	1.1598 (8)	0.8734 (5)	0.56544 (13)	0.0259 (10)	
H3	1.1272	0.9583	0.5732	0.031*	
C4	1.3332 (8)	0.8500 (5)	0.54074 (13)	0.0283 (11)	
H4	1.4162	0.9193	0.5313	0.034*	
C5	1.3830 (9)	0.7266 (5)	0.53020 (14)	0.0304 (11)	
H5	1.5014	0.7107	0.5137	0.036*	
C6	1.2612 (9)	0.6257 (5)	0.54353 (13)	0.0289 (10)	
H6	1.2966	0.5406	0.5364	0.035*	
C7	1.0871 (8)	0.6485 (4)	0.56740 (12)	0.0225 (10)	
H7	1.0023	0.5791	0.5762	0.027*	
C8	0.7140 (9)	0.9151 (4)	0.65602 (14)	0.0266 (11)	
H8A	0.6150	0.9651	0.6396	0.032*	
H8B	0.7578	0.9698	0.6780	0.032*	
C9	0.5640 (8)	0.7038 (5)	0.63773 (14)	0.0282 (11)	
H9A	0.5098	0.6214	0.6479	0.034*	
H9B	0.4556	0.7391	0.6198	0.034*	
C10	0.5989 (8)	0.7970 (4)	0.67141 (12)	0.0210 (9)	
C11	0.3785 (8)	0.8368 (5)	0.68698 (14)	0.0287 (11)	
H11A	0.2913	0.8713	0.6655	0.034*	
H11B	0.3045	0.7608	0.6975	0.034*	
C12	0.7350 (8)	0.7357 (5)	0.70299 (13)	0.0300 (11)	
H12A	0.8744	0.7098	0.6920	0.036*	
H12B	0.7618	0.7987	0.7238	0.036*	

### Atomic displacement parameters (Å^2^)

**Table:**

	*U*^11^	*U*^22^	*U*^33^	*U*^12^	*U*^13^	*U*^23^
O1	0.0287 (17)	0.0241 (16)	0.0261 (15)	−0.0100 (16)	0.0061 (15)	−0.0039 (13)
O2	0.0310 (18)	0.0194 (15)	0.0310 (16)	−0.0038 (16)	0.0109 (15)	−0.0052 (13)
O3	0.045 (2)	0.039 (2)	0.039 (2)	0.001 (2)	0.011 (2)	−0.0089 (17)
O4	0.046 (2)	0.057 (3)	0.049 (2)	0.018 (2)	0.018 (2)	0.031 (2)
C1	0.027 (2)	0.020 (2)	0.020 (2)	−0.002 (2)	0.0019 (18)	0.0012 (17)
C2	0.020 (2)	0.026 (2)	0.0188 (19)	0.0006 (19)	−0.0012 (17)	−0.0025 (18)
C3	0.032 (3)	0.025 (2)	0.0204 (19)	−0.001 (2)	0.000 (2)	−0.0016 (18)
C4	0.025 (2)	0.037 (3)	0.024 (2)	−0.008 (2)	0.0047 (19)	0.000 (2)
C5	0.024 (2)	0.042 (3)	0.025 (2)	0.000 (2)	0.006 (2)	−0.006 (2)
C6	0.031 (3)	0.030 (2)	0.025 (2)	0.003 (2)	0.000 (2)	−0.0081 (19)
C7	0.030 (2)	0.016 (2)	0.0219 (19)	−0.0002 (19)	0.000 (2)	0.0024 (16)
C8	0.038 (3)	0.0165 (19)	0.025 (2)	−0.006 (2)	0.007 (2)	−0.0024 (18)
C9	0.027 (3)	0.025 (2)	0.032 (2)	−0.009 (2)	0.008 (2)	−0.0056 (19)
C10	0.023 (2)	0.0186 (19)	0.0213 (19)	0.0015 (19)	0.0029 (18)	0.0017 (16)
C11	0.023 (2)	0.033 (3)	0.031 (2)	0.006 (2)	0.000 (2)	0.000 (2)
C12	0.025 (2)	0.042 (3)	0.023 (2)	0.014 (2)	0.004 (2)	0.006 (2)

### Geometric parameters (Å, °)

**Table:**

O1—C1	1.403 (5)	C5—H5	0.9500
O1—C8	1.443 (6)	C6—C7	1.388 (7)
O2—C1	1.409 (5)	C6—H6	0.9500
O2—C9	1.441 (6)	C7—H7	0.9500
O3—C11	1.421 (6)	C8—C10	1.526 (6)
O3—H3O	0.8400	C8—H8A	0.9900
O4—C12	1.432 (7)	C8—H8B	0.9900
O4—H4O	0.8399	C9—C10	1.534 (6)
C1—C2	1.512 (6)	C9—H9A	0.9900
C1—H1	1.0000	C9—H9B	0.9900
C2—C3	1.382 (7)	C10—C12	1.526 (6)
C2—C7	1.392 (6)	C10—C11	1.539 (7)
C3—C4	1.403 (7)	C11—H11A	0.9900
C3—H3	0.9500	C11—H11B	0.9900
C4—C5	1.377 (7)	C12—H12A	0.9900
C4—H4	0.9500	C12—H12B	0.9900
C5—C6	1.381 (8)		
			
C1—O1—C8	110.1 (4)	O1—C8—H8A	109.3
C1—O2—C9	110.1 (4)	C10—C8—H8A	109.3
C11—O3—H3O	109.0	O1—C8—H8B	109.3
C12—O4—H4O	108.9	C10—C8—H8B	109.3
O1—C1—O2	111.3 (3)	H8A—C8—H8B	108.0
O1—C1—C2	108.9 (4)	O2—C9—C10	110.6 (4)
O2—C1—C2	108.8 (4)	O2—C9—H9A	109.5
O1—C1—H1	109.3	C10—C9—H9A	109.5
O2—C1—H1	109.3	O2—C9—H9B	109.5
C2—C1—H1	109.3	C10—C9—H9B	109.5
C3—C2—C7	119.5 (4)	H9A—C9—H9B	108.1
C3—C2—C1	119.7 (4)	C8—C10—C12	109.0 (4)
C7—C2—C1	120.8 (4)	C8—C10—C9	108.6 (4)
C2—C3—C4	120.0 (5)	C12—C10—C9	110.7 (4)
C2—C3—H3	120.0	C8—C10—C11	109.1 (4)
C4—C3—H3	120.0	C12—C10—C11	111.4 (4)
C5—C4—C3	120.0 (5)	C9—C10—C11	108.0 (4)
C5—C4—H4	120.0	O3—C11—C10	111.3 (4)
C3—C4—H4	120.0	O3—C11—H11A	109.4
C4—C5—C6	120.2 (5)	C10—C11—H11A	109.4
C4—C5—H5	119.9	O3—C11—H11B	109.4
C6—C5—H5	119.9	C10—C11—H11B	109.4
C5—C6—C7	120.1 (5)	H11A—C11—H11B	108.0
C5—C6—H6	120.0	O4—C12—C10	110.6 (4)
C7—C6—H6	120.0	O4—C12—H12A	109.5
C6—C7—C2	120.3 (4)	C10—C12—H12A	109.5
C6—C7—H7	119.9	O4—C12—H12B	109.5
C2—C7—H7	119.9	C10—C12—H12B	109.5
O1—C8—C10	111.4 (4)	H12A—C12—H12B	108.1
			
C8—O1—C1—O2	−64.2 (5)	C1—C2—C7—C6	177.4 (4)
C8—O1—C1—C2	175.9 (4)	C1—O1—C8—C10	56.9 (5)
C9—O2—C1—O1	65.2 (5)	C1—O2—C9—C10	−58.0 (5)
C9—O2—C1—C2	−174.9 (4)	O1—C8—C10—C12	70.6 (5)
O1—C1—C2—C3	−50.6 (5)	O1—C8—C10—C9	−50.1 (5)
O2—C1—C2—C3	−172.1 (4)	O1—C8—C10—C11	−167.6 (4)
O1—C1—C2—C7	132.3 (4)	O2—C9—C10—C8	50.6 (5)
O2—C1—C2—C7	10.9 (6)	O2—C9—C10—C12	−69.0 (5)
C7—C2—C3—C4	0.8 (7)	O2—C9—C10—C11	168.7 (4)
C1—C2—C3—C4	−176.2 (4)	C8—C10—C11—O3	−57.8 (5)
C2—C3—C4—C5	−1.4 (7)	C12—C10—C11—O3	62.5 (5)
C3—C4—C5—C6	0.7 (8)	C9—C10—C11—O3	−175.6 (4)
C4—C5—C6—C7	0.5 (8)	C8—C10—C12—O4	178.6 (4)
C5—C6—C7—C2	−1.0 (7)	C9—C10—C12—O4	−62.0 (5)
C3—C2—C7—C6	0.3 (7)	C11—C10—C12—O4	58.2 (5)

### Hydrogen-bond geometry (Å, °)

**Table:**

*D*—H···*A*	*D*—H	H···*A*	*D*···*A*	*D*—H···*A*
O4—H4O···O3^i^	0.84	2.19	2.644 (6)	114

Symmetry codes: (i) *x*+1/2, *y*−1/2, *z*.
